# Usability Assessment of Body Controlled Electric Hand Prostheses: A Pilot Study

**DOI:** 10.3389/fnbot.2021.683253

**Published:** 2021-11-05

**Authors:** Sasha B. Godfrey, Cristina Piazza, Federica Felici, Giorgio Grioli, Antonio Bicchi, Manuel G. Catalano

**Affiliations:** ^1^Soft Robotics for Human Cooperation and Rehabilitation, Center for Robotics and Intelligent Systems, Istituto Italiano di Tecnologia, Genoa, Italy; ^2^Assistive and Restorative Technology Laboratory, Rehabilitation Medicine Research Center, Mayo Clinic, Rochester, MN, United States; ^3^Department of Informatics and Munich Institute of Robotics and Machine Intelligence, Technical University of Munich, Munich, Germany; ^4^Centro “E. Piaggio” and Dipartimento di Ingegneria Informatica, University of Pisa, Pisa, Italy

**Keywords:** prosthetic hand, myoelectric control, body-powered prostheses, prosthetic control, soft robotics

## Abstract

Poly-articulated hands, actuated by multiple motors and controlled by surface myoelectric technologies, represent the most advanced aids among commercial prostheses. However, simple hook-like body-powered solutions are still preferred for their robustness and control reliability, especially for challenging environments (such as those encountered in manual work or developing countries). This study presents the mechatronic implementation and the usability assessment of the SoftHand Pro-Hybrid, a family of poly-articulated, electrically-actuated, and body-controlled artificial hands, which combines the main advantages of both body-powered and myoelectric systems in a single device. An assessment of the proposed system is performed with individuals with and without limb loss, using as a benchmark the SoftHand Pro, which shares the same soft mechanical architecture, but is controlled using surface electromyographic sensors. Results indicate comparable task performance between the two control methods and suggest the potential of the SoftHand Pro-Hybrid configurations as a viable alternative to myoelectric control, especially in work and demanding environments.

## 1. Introduction

Upper limb loss is disproportionately found in developing countries with trauma and war as the most common causes (World Health Organization, 2004). While disease is also a frequent cause, upper limb loss globally tends to affect a younger, working-age population (van der Sluis et al., 2009). It is therefore important that prosthetic solutions take this population into account by being economically accessible and robust to strenuous use or hostile environments. However, a 1985 study found that 75% of persons with amputation (upper, lower, and multiple) change occupation group when they return to the work-force post-amputation, moving from machining, processing, fabrication, and construction to service, clerical, and sales (Millstein et al., 1986). Additionally, only 21% returned to their pre-amputation job and more than half noted negative repercussions on career potential following amputation (Millstein et al., 1986). A recent literature review (Darter et al., 2018) found that data on returning to work post-amputation varies greatly (48–89% of individuals) but that returning to one's previous position continues to be rare and less frequent for manual rather than office work.

**Figure 1:**
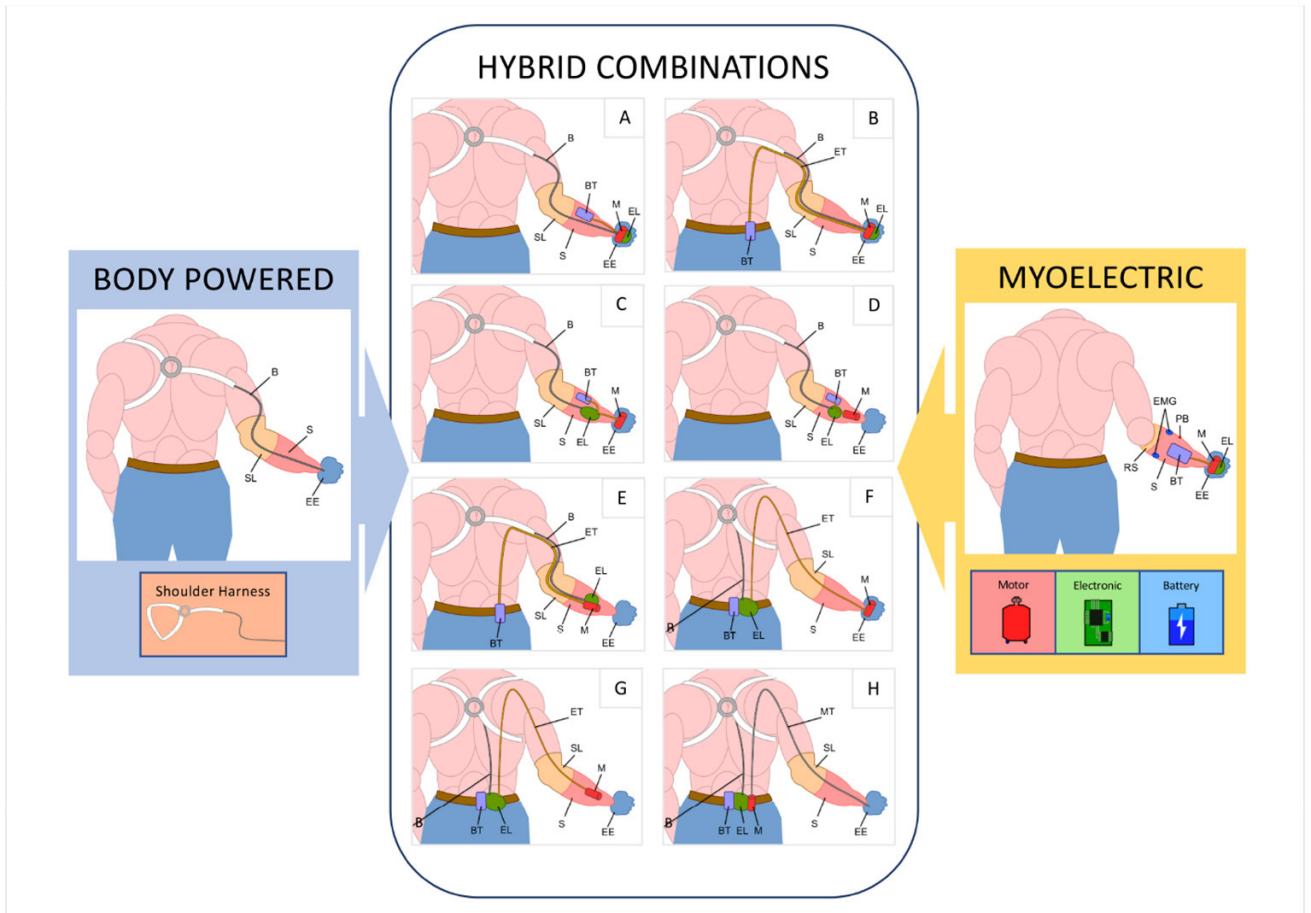
The typical set-up of a body-powered prosthesis (using a figure-of-nine harness) is shown on the left and a typical myoelectric prosthesis on the right. The input and terminal device components highlighted in the inset are combined in hybrid systems. Main components: EE, Prosthetic Device; S, Socket; RS, Rigid Socket; SL, Soft Socket Liners; B, Cable Control System; EMG, Surface Electromyographic Sensors; M, Motors; EL, Electronic Boards; BT, Battery Pack; PB, Power Button; ET, Electrical Transmission Cable; MT, Flexible Transmission Shaft.

Active prosthetic solutions are either body-powered or electric. Most of the former are controlled using a shoulder harness that encompasses one or both shoulders (figure-of-nine or figure-of-eight, respectively) depending on the amputation(s), see Figure 1. Most of the latter are myoelectrical controlled, that is using muscle signals in the residual limb. Myoelectric devices are typically anthropomorphic in appearance if not in structure: most devices have a hand-shaped glove or shell covering a tri-digit structure. The newest devices, however, have five fingers and multiple motors to enable various postures. While the former is relatively simple to control, they provide only a single, rigid, C-shaped grasp. The latter, in contrast, offer more grasp modalities but place a higher cognitive burden on the user (Kuiken et al., 2016). Along with control complexity, weight tends to increase with myoelectric prosthetic complexity, due in part to the use of multiple motors (Belter et al., 2013), and robustness tends to decrease. In contrast, body-powered prostheses are most typically split-hook models and thus are not anthropomorphic. Some specific domains show better performance by one type of prosthesis over the other (Carey et al., 2017), e.g., myoelectric hands tend to be more accepted for low-intensity work, while their drawbacks, in general, render them less desirable for use in manual tasks in home and work environments and in resource-poor areas, which represent tough testing grounds for a prosthetic system. Conversely, body-powered hooks are the most used aids in such contexts. As such, many prosthesis users have more than one prosthetic device, choosing between them based on activity or setting, for example preferring one for work environments and another for social situations (Millstein et al., 1986; Dakpa and Heger, 1997).

Each type has its advantages and disadvantages, and although commercial and research innovations tend toward highly-sophisticated myoelectric devices, many individuals continue to use body-powered systems (Biddiss and Chau, 2007b; Østlie et al., 2012), which have seen only minor improvements in the last century (Hashim et al., 2018). Sophisticated myoelectric technologies may be difficult to control or lack reliability and may not provide adequate support across all activities of daily living. The latter aspect is also highlighted by the outcomes of international competitions such as the Cybathlon - Powered Arm Prosthetic Race (Riener, 2016). In both editions (Cybathlon 2016 and Cybathlon 2020) approaches aiming at simple body-powered designs proved notable benefits and outperformed all myoelectric prostheses in the competition. This is not to say that one prosthesis type is superior to another. There is insufficient evidence to draw such a conclusion and ultimately the user must decide based on need, access, and other personal factors (Carey et al., 2015). Indeed, Østlie et al. (2012) surveyed 181 prosthesis users and found roughly 30% used a body-powered device as their primary prosthesis and roughly 34% used a myoelectric device. Of those users listing a secondary device, roughly 36% used a body-powered device and 27% a myoelectric one.

Looking more closely at these advantages and disadvantages, body-powered prostheses tend to be more robust and lighter than their myoelectric counterparts, with different materials available (aluminium, steel, etc.) to balance robustness and weight to suit the needs of the user. Furthermore, the shoulder harness provides a straight-forward and easy-to-use control method with limited sensory feedback (Brown et al., 2017), at the cost of applying pressure to the axilla, which can cause discomfort or even damage to the brachial plexus over time (Fryer, 1992). Users of myoelectric devices tend to rely on visual feedback to guide movements; however, the sound of the motor is also used to inform prosthetic control (Antfolk et al., 2013). In terms of comfort, myoelectric systems require a rigid interface between the socket and the residual limb to ensure adequate contact with surface electromyographic sensors (sEMG), while body-powered systems can employ more comfortable soft socket liners, and the contact area with the residual limb can be significantly increased. Moreover, performance of sEMG drops severely when impurities, such as dirt or sweat, interpose between the sEMG and the underlying skin; this problem can be mitigated at least in part with the use of solutions such as linear transducers, switches, and other biomechanical control methods (Childress, 1992; Muzumdar, 2004). Body-powered prosthesis function does not suffer from these aspects.

This work explores the concept of a “hybrid” configuration that aims to feature robust and intuitive control in a prosthesis that is both resilient to harsh environments, highly functional, and anthropomorphic, thanks to the combination of the main advantages of both body-powered and myoelectric systems in a single device. As presented in Figure 1, a myoelectric hand prosthesis requires three main additional components compared to a body-powered prosthesis: a motor to actuate the device, an electronic board to control it, and a battery to power it, all generally located within the hand and socket. The approach proposed has the advantage of enabling multiple solutions through the placement of the electro-mechanical components in three possible locations: on the hand, in the socket, or on the body of the user. Considering different solutions among these, the designer can thus create a class of devices suitable for different applications.

Although current commercial prostheses are rigid, novel trends in robotic research are moving the state of the art of artificial hands in a different direction, which includes soft materials and structures and simpler actuation mechanisms (Piazza et al., 2019). Soft robotics may present a particular advantage in challenging environments by naturally being more robust to collisions and similar mis-use (Negrello et al., 2020). In Godfrey et al. (2018), we proposed the SoftHand Pro, a myoelectric prosthetic device whose movement is based on neuroscientific principles of hand joint coordination, or synergies, in line with the proposal above. Despite good results in terms of grasping capabilities, ease of use, and user acceptance (Godfrey et al., 2018), the SoftHand Pro maintains those drawbacks directly related to the use of sEMG sensors (e.g., sensitivity to dirt and dust, socket interface, costs). Moreover, functionality of myoelectric solutions is dependent on many constraints, not only connected with the technology itself but also related to clinical aspects (i.e., insufficient muscle activation in the residual limb). For these reasons, in Piazza et al. (2017), we presented the concept of the SoftHand Pro-Hybrid (SHPH), to combine the easy-to-use control of a body-powered prosthesis with the power available from an electric terminal device. As introduced in the detailed analysis of Piazza et al. (2017), considering all possible combinations of components and locations, 8 potential solutions were isolated (refer to Table 1, for details), and one of them was briefly evaluated with only one amputee subject (an expert user of a body-powered hook). The work presented in Piazza et al. (2017) sketches the main ideas behind the platform and shows the main technical advantages of such an approach: simple control of the prosthetic hand while retaining high grip power and providing a high level of robustness, adaptivity, and resilience. However, it does not provide any clinical assessment of the platform or any information about the mechatronic designs that can be used to build all the possible solutions.

**Table 1:** Summary of hybrid component locations for 8 feasible solutions (Piazza et al., 2017) and, for reference, myoelectric solution (BM) component location (Godfrey et al., 2018).

**SOL**	**HAND**	**SOCKET**	**EXT BOX**
	** 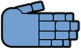 **	** 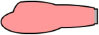 **	** 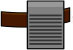 **
A	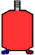 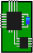	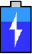	
B	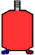 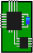		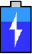
C	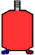	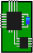 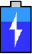	
D		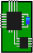 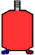 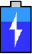	
E		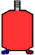 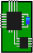	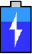
F	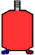		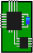 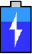
G		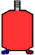	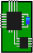 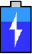
H			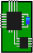 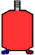 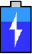
BM	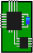 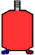	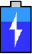	-

The work presented herein has as its main goal to assess the usability of a hybrid system with multiple subjects (not expert body-powered users), through the adoption of standard clinical tests, and make a comparison with a system, based on the same architecture and used as a benchmark, but activated by conventional myoelectric control (the SoftHand Pro). Given the lack of literature on specific tests to assess prostheses in work and challenging environments, we selected the ACMC test and the System Usability Scale (SUS) as our main outcome measures. The former because it takes in consideration several aspects of manipulation (e.g., the releasing or the holding phase) in the context of, but independent from, everyday tasks, and the latter because it is a standard questionnaire used to evaluate technological devices. The results of this investigation suggest that hybrid solutions can be a valid alternative to myoelectric control, e.g., in situations that require high grip power, grasp versatility and resilience or depending on user preferences. Specifically, hybrid solutions may be more suitable for working activities and challenging environments, where the use of sEMG sensors, that can be sensitive to sweat or socket alignment, increase the overall complexity. The study extends the work in Piazza et al. (2017) exploring the usability of two of the solutions presented in that work. Furthermore, it provides a first insight into the possibility of using the SoftHand architecture to make a direct comparison between myoelectric and body-power control modes. Indeed, few studies address this topic (Edelstein and Berger, 1993; Carey et al., 2009), which is of great relevance to understanding which control modes are best suited to a user or use-case. In the opinion of the authors,these types of comparisons could help advance the research field and warrant deeper investigation in future work. To achieve these goals, we proceed as follows. Sections 2.1, 2.2, and 2.3 present the mechanical implementation of the hybrid and myoelectric solutions, while section 2.4 discusses pros and cons of each layout, with respect to a set of specifications and indications that comes from a detailed analysis of surveys and studies available in the state of art. Then, sections 2.5 and 2.6 present the assessments selected for this analysis and the protocol performed with individuals with and without limb loss. Results are presented in section 3 and discussed in section 4.

## 2. Materials and Methods

### 2.1. SoftHand Architecture

The mechanical structure of the terminal devices considered in this work is based on the architecture of the Pisa/IIT SoftHand (Catalano et al., 2014), from which both the myoelectrically controlled and the hybrid body controlled prosthetic hands are derived. The architecture presents an anthropomorphic shape with 19 DoFs. Each finger consists of a group of rolling joints connected by elastic ligaments. The elastic bands, fixed on either side of the joint, make the system soft and safe and allow the hand to automatically return to its correct configuration, i.e., after severe dislocations. The transmission system uses one tendon that runs through the entire hand in two levels of pulleys, giving adaptivity to the overall system without a differential gear mechanism. The soft robotic mechanical design gives the hand an overall robustness with the capability of adapting its closure to the shape of objects. Phalanges and structural parts of the palm are crafted using high-performance thermoplastic materials (nylon reinforced with 35% of glass fibres), and are produced using injection molding techniques, enabling a sizable reduction of weight and costs. Such technological solutions allow the possibility of working in harsh and dangerous environments (for more details please refer to Piazza et al., 2017; Godfrey et al., 2018; Mura et al., 2018; Negrello et al., 2020). As an example of these capabilities, photo-sequence in **Figure 10** shows one of the subjects enrolled in this study performing a grasping task in an underwater setting.

### 2.2. Body Controlled SoftHand: Concept and Development

The hybrid solutions adopt the structural architecture described in section 2.1 and integrate a Hosmer body-powered wrist to interface with the socket, enabling prono-supination movements through manual wrist rotation. The idea of a hybrid configuration can be realized in different ways. In our approach, the hybrid system uses an electromechanical lever to translate inputs from a shoulder harness to motor control. The electromechanical lever consists of a linear mechanism and spur gear. The position is read by a single encoder that transmits signals to the motor through the electronic board to command hand opening/closing.

**Figure 2:**
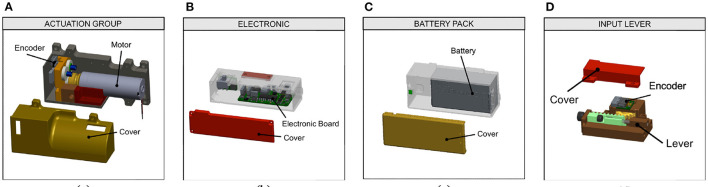
CAD model of the three main mechanical subsystems: **(A)** the actuation group, **(B)** the electronic, and **(C)** the battery pack. The hybrid devices use an input lever **(D)** to translate the shoulder movement into hand commands.

The amount of shoulder excursion required to correctly operate the SHPH (i.e., to yield full opening/closure and switch between control modalities) is modifiable to balance comfort, ease-of-use, sensitivity, and resolution. The benefits of this mechanism are visible already in Piazza et al. (2017). These values were adjusted for each study subject at the start of each trial and, if needed, fine-tuned during the training period. Additionally, the user can use the lever to switch between two modes, voluntary-opening (VO) and voluntary-closing (VC), (Sensinger et al., 2015) by compressing the end-stroke spring on the remote or mounted lever through additional shoulder rotation. The ability to switch between modes allows users to employ whichever they were most comfortable with, both in general and, if desired, on a task-by-task basis; for example, using VO for carrying robust and/or heavy objects and VC for handling more fragile objects or for precision grasping. The other components can be grouped into three modules, each one embedding a mechanical subsystem as shown in Figure 2:

1. Actuation Group, which includes the motor and a position sensor. In our implementation, a DCX 22S + GPX 83:1 Maxon motor is used, combined with an Austrian Microsystem Magnetic Encoder.
2. Electronic Board, equipped with a Cypress PSOC micro-controller and a daisy chain RS485 Bus and several i/o connections. The board can communicate with magnetic sensors through the SSR protocol. The custom electronic board is derived from Della Santina et al. (2017);
3. Battery Pack, used to power the hand (in our implementation, an off-the-shelf battery from Parrot AirDrone 2.0 with a capacity of 1,500 mAh).

The technical specifications of the components included in the three modules are reported in Table 2; these can be used to evaluate all eight proposed solutions. All eight solutions are practicable and can be adapted to different situations, activities or to meet user needs.

**Table 2:** Technical specifications of the three mechanical subsystems.

	**Motor Group 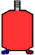 **	**Electronic 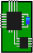 **	**Battery Pack 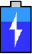 **
Weights	130 g	15 g	202 g
Dimensions	*d* = 22 mm, *l* = 80 mm	60 × 30 × 12 mm	94 × 66 × 37 mm

This concept becomes clearer if we apply a multi-variate Pareto analysis (Hwang and Yoon, 2012) to the different configurations following specific criteria. Using this method, it is possible to highlight how each configuration measures against the different criteria, which ideally should be selected and customized with the user. To give a more clear example of this concept, we choose four criteria considering the critical factors motivating device abandonment and leading consumer design priorities, as stated in the literature. From Cordella et al. (2016) it is evident how comfort, function and appearance of the prosthesis are the aspects with the highest priority for users and common to all device types. In particular (Biddiss et al., 2007) highlights how, for body-powered systems, harness comfort and weight reduction are among the main problems experienced by the users. Moreover, it is also important to take into account characteristics specific to the user, such as the level of limb loss (Biddiss and Chau, 2007a), a factor which is strictly connected to the design of the socket and to the placement of the device electro-mechanical components. Starting from these considerations, the following criteria were selected for this study:

1. Shoulder torque, directly related to system weight and placement (Cordella et al., 2016). It is calculated considering the linear distance from the shoulder to the component using standard anthropometric measurements (average data between male and female) from McConville et al. (1980); Dempster (1955) and the weights of each component. We are not considering the weight of the terminal device and socket because, for our analysis, it will be the same in all the configurations. This criterion relates both to function as well as comfort. If the shoulder torque is excessively high, the hand will be more difficult to operate, as it will require more strength. Additionally, shoulder torque contributes to repetitive use strain and injury, typically to the brachial plexus, as mentioned above (Fryer, 1992);
2. Number of elements to wear, an important consideration in terms of comfort (Cordella et al., 2016) as well as appearance. Wearing multiple components can render the system more bulky and less aesthetically pleasing as well as potentially making the system more challenging to don and doff. The elements considered in this analysis are hand, socket and an external box. Note: as the hand and socket are necessary for all solutions, this criterion refers primarily to the external box;
3. Cable length, to connect each subsystem (both electric and driving cables) and calculated considering the distance the cables traverse across the arm and/or back following standard anthropometric measurements from McConville et al. (1980); Dempster (1955). As discussed above, comfort in term of cables is highlighted as an important aspect for body-powered users (Biddiss et al., 2007). In addition to comfort, cabling can affect function in terms of mechanical feasibility, as some of the user's shoulder effort is lost along the flexible transmission shaft. Additionally, cabling may affect both appearance and aesthetics depending on cable routing and the user's ability to hide cables under clothing, if desired;
4. Total volume occupied by the components included in the socket, calculated considering the volume of each electromechanical subsystem for each case. This aspect should not be underestimated as it relates to limb length (important for function and aesthetics) and device feasibility, depending on residual limb length and especially in the case of users with distal amputation (Biddiss and Chau, 2007a).

**Figure 3:**
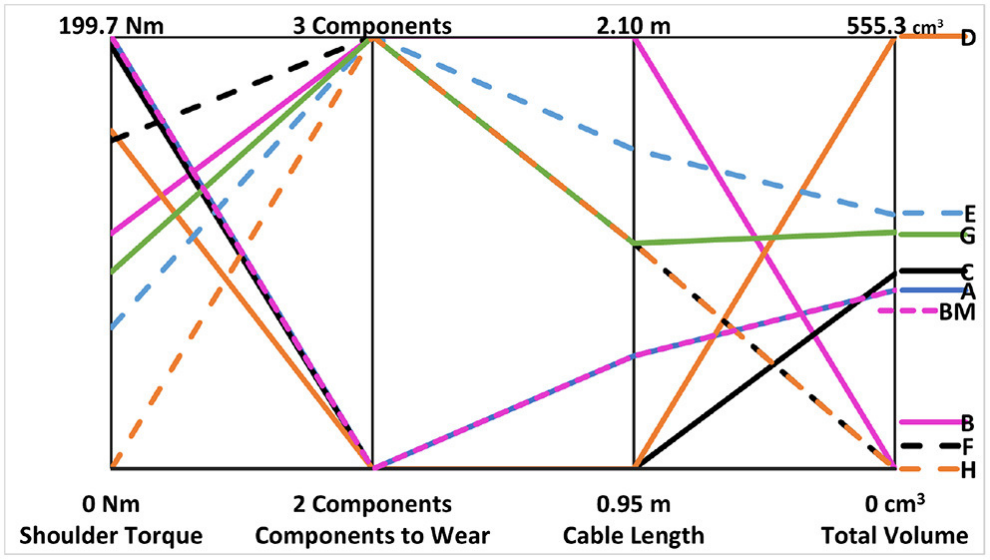
Results of the Pareto analysis applied to the 8 hybrid configurations (preliminary presented in Piazza et al., 2017), considering 4 indices: (1) the shoulder torque, (2) the number of elements to wear, (3) the cable length and (4) the total volume occupied by the components included in the socket. NB: values for the benchmark are presented by the dashed, magenta line labeled “BM”.

The metrics for each criterion for the hybrid configurations are shown in Table 3, while the results of the analysis are presented in Figure 3. For each criterion, a smaller number is preferable. The goal of the Pareto analysis is to find the ideal solution considering multiple criteria, thus configurations were considered that minimized the most criteria. It should be noted that the most criteria minimized by any configuration were two; no configuration minimized three or four criteria. From the analysis, Configurations C and D minimized both “Components to Wear” and “Cable Length”, while Configuration H minimizes “Shoulder Torque” and “Total Volume”. Solutions where all components are integrated into the hand and socket (Configuration A, C, and D) provide the advantage of being less cumbersome and supplying the components with at least limited protection against environmental factors such as dust or liquid. These solutions, however, depend on the length of the residual limb. For longer limb lengths, more components can be placed on the body (such as Configurations F-H), providing additional protection against environmental factors, possibly even rendering the system waterproof at the level of the hand or arm. Finally, for scenarios requiring increased grasp strength, solutions such as those in Configurations G or H could be used with more powerful motors. Considering the results of the analysis and the features of the configurations themselves, we chose to develop two different solutions. Configurations C and D minimize the same two criteria, as mentioned above. Configuration D creates 25% less shoulder torque while Configuration C requires less than half the total volume. As mentioned earlier, total volume is an essential aspect of device feasibility, usability, and acceptance and thus Configuration C was chosen over D. Configuration C was developed alongside Configuration H, as they provide very different theoretical advantages to the user.

**Table 3:** Data of shoulder torque, components to wear, cable length, and total volume criteria for each hybrid configuration and benchmark.

**SOL**	**Shoulder** **torque (Nm)**	**Comp. to** **wear**	**Cable** **length (m)**	**Total vol.** **(cm^3^)**
A	199.650	2	1.250	229.548
B	108.750	3	2.100	0
C	195.150	2	0.950	251.148
D	156.150	2	0.950	555.254
E	65.250	3	1.800	325.706
F	151.500	3	1.550	0
G	90.900	3	1.550	304.106
H	0	3	1.550	0
BM	199.650	2	1.250	229.548

### 2.3. Myolectrically Controlled SoftHand

The myoelectrically controlled SoftHand Pro, used as a benchmark, adopts the structural architecture described in section 2.1, and is actuated by a DCX 22S + GPX 83:1 Maxon motor (as in the hybrid configurations) mounted on the dorsal side of the hand. This hand shares the same electronic board of the hybrid hands, but can read and elaborate EMG signals provided by a pair of sensors (13E200=60, OttobockGmbH, Germany). Different standard controllers are available in the board; the one adopted in this study is based on an integral control of myoelectric signals which command the reference velocity of the motor (for more details see Godfrey et al., 2018). Finally, the hand is equipped with a standard Ottobock Quick Disconnect Wrist, enabling wrist prono-supination movements through manual wrist rotation, comparable to the Hosmer device used for the hybrid configurations.

### 2.4. Experimental Setup

**Figure 4:**
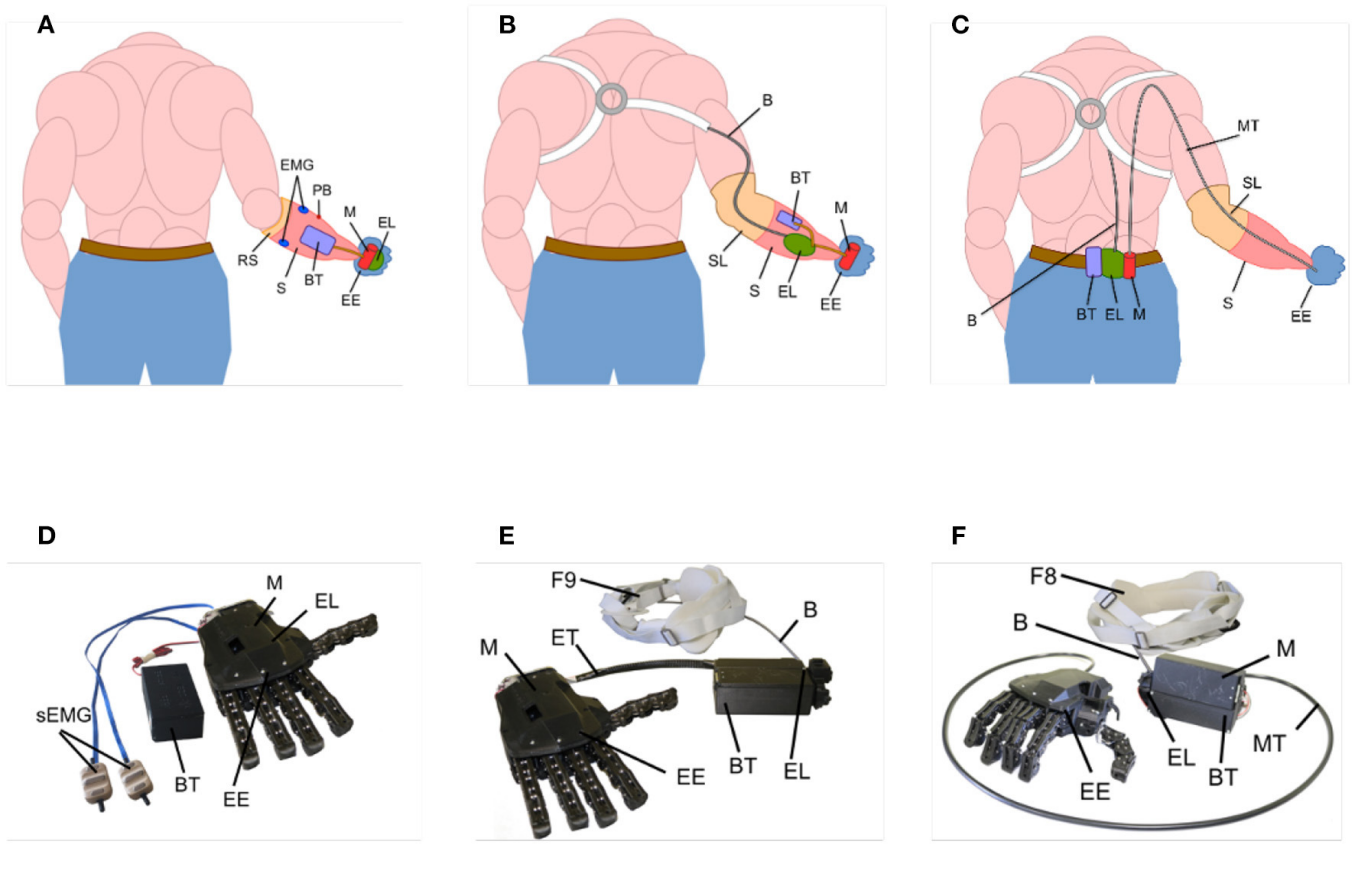
Schematic placement (**A–C**; Benchmark, Compact, and Hardy Configurations, respectively) of the components of the configurations tested in this study on the hand, socket, and waist of the user and pictures **(D–F)** of the physical devices used. EE, Prosthetic Device; S, Socket; RS, Rigid Socket; SL, Soft Socket Liners; B, Cable Control System; EMG, Surface Electromyographic Sensors; M, Motors; EL, Electronic Boards; BT, Battery Pack; PB, Power Button; BT, Battery Pack; ET, Electrical Transmission Cable; MT, Flexible Transmission Shaft; F8, Figure-of-eight Harness; F9, Figure-of-nine Harness.

Among the eight possible hybrid solutions (please refer to Table 1), the solutions which best fit the selected criteria for our analysis (extracted from the literature) were solutions C and H. These two solutions were dubbed the Compact and Hardy Configurations, solutions C and H, respectively. The SoftHand Pro (Godfrey et al., 2018), was used as a benchmark. An overview of the three solutions used in the experimental section is presented in Figure 4, highlighting the main components:

- *Benchmark Configuration*, myoelectrically controlled using 2 surface EMG sensors included directly in the socket. Motor and electronics are embedded directly in the palm of the hand.
- *Compact Configuration*, this hybrid configuration uses a figure-of-nine harness as input control. The motor is embedded in the hand, while the electronics, lever, and battery are integrated in the socket/forearm of the user.
- *Hardy Configuration* displaces all three electromechanical components to the waist. In this hybrid configuration, the actuation lever is connected to a figure-of-eight harness. A steel Bowden cable goes from the motor group to the hand-winding system, to operate hand opening and closing.

Broadly speaking, the Compact Configuration has the main components embedded in the hand and socket, which makes the system more integrated but increases the weight of the distal part and reduces resistance to liquids. In the Hardy solution, the electromechanical components are placed on the body of the user, which makes the whole system bulkier and less aesthetically pleasing but can be very effective for work environments because the total weight of the system is distributed along the body of the user and the terminal device is capable of interacting with dangerous environmental factors such as dust or liquids (see **Figure 10**). The Benchmark Configuration, similar to the Compact one, has the electromechanical components distributed in the hand and socket. This configuration is myoelectrically controlled so no cables are passing along the body of the user. However, the sEMG sensors are sensitive to environmental factors (i.e., liquid, dust, etc.) or impurities (i.e., dirt or sweat), which decreases the suitability of this system in working environments.

### 2.5. Assessment Tools

The hybrid configurations were validated with standard clinical and technology assessments (ACMC, BBT, SUS) to evaluate the strengths and weaknesses of the proposed solutions. The Assessment of Capacity for Myoelectric Control (ACMC) is a standard observational clinical test able to assess the ability to control a prosthetic hand in a daily living task (Hermansson et al., 2005). The rater may choose one among six validated tasks for the testee to perform, which reproduce hobbies or activities of daily living. The tasks were designed to feature similar types of movements in different contexts and to be interchangeable. Each item is an observable prosthetic hand movement on its own or in relation to other body parts and is scored from zero (incapable) to three (extremely capable). An algorithm (accessible online) is used to convert the raw score to ACMC logits, ranging from 0 to 100 with 100 representing ideal performance and prosthetic control. Among the different outcome measures available in the literature, the ACMC is unique, to the authors' knowledge, in breaking down tasks or actions into sub-movements of grasping, holding, releasing, and repetitive movements (see Table 8). Many tests were considered when designing the protocol, including the AM-ULA (Resnik et al., 2013) and the SHAP (Light et al., 2002). The former is rated by an experienced rater, usually an occupational or physical therapist, on the ability to perform a set of 18 ADLs and the quality of that performance, including smoothness of movements, speed, appropriateness and precision of grasp, etc. The latter is scored by time to completion of various tasks including grasping different light and heavy forms and completing ADLs; the rating is given by an algorithm that compares these times to a benchmark of able-bodied individuals. To enable comparison to a benchmark set of data, the SHAP protocol must be adhered to strictly, sometimes impeding a testee's typical way of approaching a particular task. While all three tests have strengths and weaknesses, ultimately, the ACMC was chosen as our primary outcome measure because, at this stage of device development, it allowed a better understanding of the detailed movement stages required to complete a task rather than task completion itself and focused specifically on control capacity. The Box and Blocks Test (BBT) is a standard clinical test to evaluate unilateral gross manual dexterity (Desrosiers et al., 1994). In the BBT, subjects are asked to move as many blocks as possible in 1 min from one compartment of a box to the other, with a vertical divider between them. The number of blocks carried over the partition is used to score the test. BBT was designed to measure hand performance in patients with neuromuscular disorders; it is often also used in prosthetic evaluations and is valid in this context (Resnik and Borgia, 2012). The System Usability Scale (SUS) is a simple, effective, and widely exploited tool for measuring the usability of a device (Brooke, 1996); it does not serve a diagnostic function nor is it limited to clinical applications. It is a 10-item questionnaire with five response options, ranging from “strongly agree” to “strongly disagree”. The subject's scores for each question are converted to a new number between 0 and 4 based on whether the question was framed in a positive light (e.g., “I think that I would like to use this system frequently.”) or a negative one (e.g., “I found the system unnecessarily complex.”). The ten ratings are then added together and multiplied by 2.5 to convert the original cumulative score of 0–40 to 0–100.

### 2.6. Experimental Protocol

**Figure 5:**
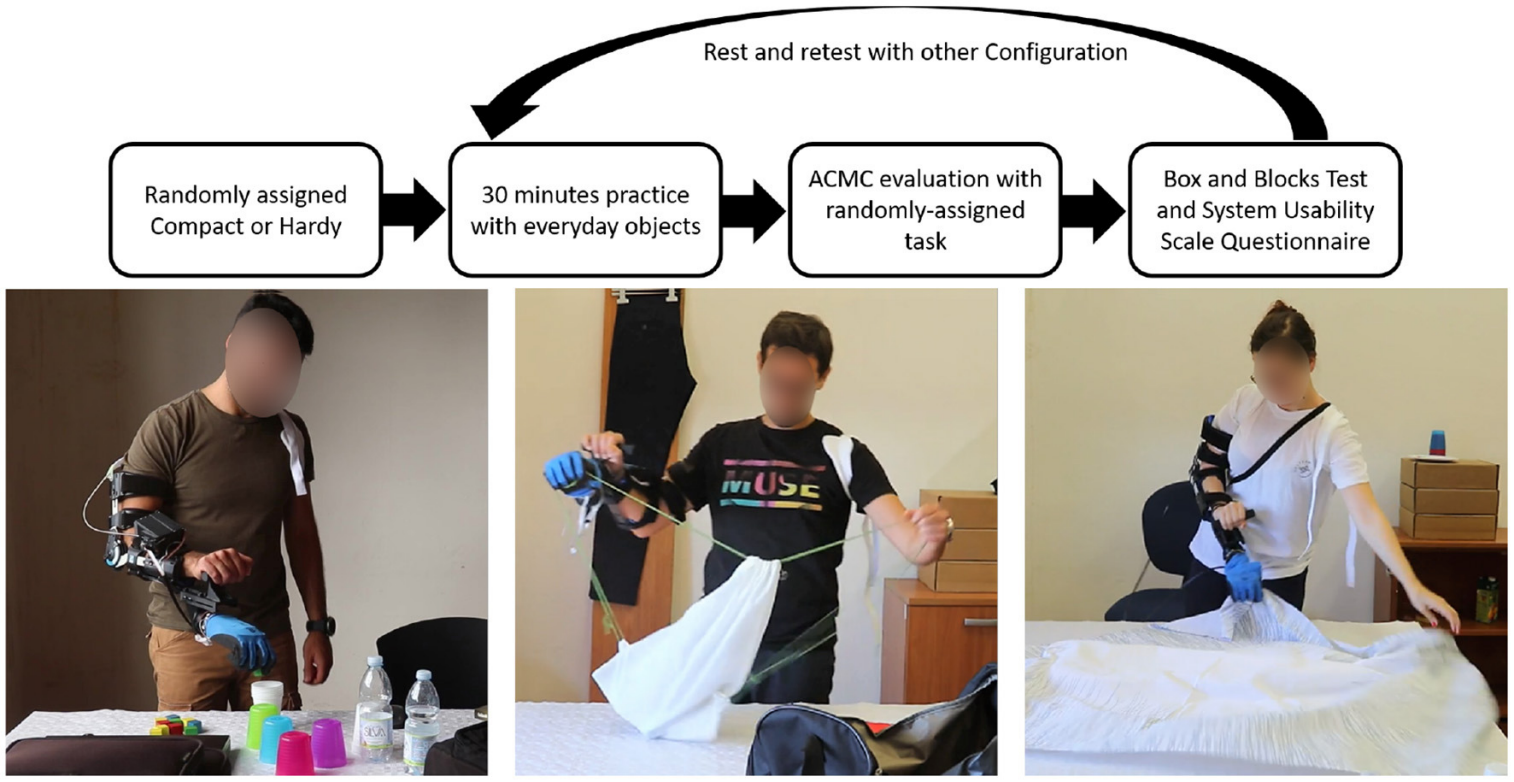
**Top**: Flow-chart of the experimental protocol. **Bottom**: Intact-limb subjects practicing with everyday objects (left) and testing the two Hybrid Configurations during tasks of daily living as part of a standardized clinical test: SHPH-C, ACMC “packing” task (middle) and SHPH-H, ACMC “setting the table” task (right).

The research was performed under the oversight of the local ethics committee (Comitato Etico di Area Vasta Nord Ovest, CEAVNO), protocol number 1072. Informed Consent was obtained from all subjects. An open, crossover, experimental study was performed. The study was composed of two phases, one for each population, performed first with limb-intact subjects and subsequently with subjects with limb loss. The protocol was identical for both subject populations. Experiments with limb-intact subjects were performed first, to provide a preliminary evaluation of the usability of the two SHPH configurations, which had never undergone testing before. Then, tests with subjects with limb loss followed, aimed at verifying limb-intact results and highlighting the advantages and disadvantages of the tested devices in comparison with a standard myoelectric configuration (SHP). Limb-intact subjects wore a forearm adapter that placed the SHPH under their natural hand. Furthermore, these subjects wore an arm brace (Innovator X Post-Op Elbow, Ossur) that restricted pronation/supination to more closely mimic the limitation or absence of this DOF in subjects with limb loss (see Figure 5). The latter subjects wore the SHP and SHPH on their typical socket or a socket made specifically for this study by a certified prosthetist.

Inclusion criteria for limb-intact subjects were 18−80 years of age, with normal motor and cognitive function, right-handedness, and able to understand the experimental procedures and give informed consent. For subjects with limb loss, inclusion criteria were 18–80 years of age, transradial difference, ability to understand the experimental procedures and give informed consent. Ten subjects matching the inclusion criteria, 7 males and 3 females, ranging from 25 to 32 years old, were included in the limb-intact group. The limb loss group comprised 3 subjects, 2 females and 1 male all with left transradial difference. Demographic information for the subjects with limb loss can be found in Table 4.

**Table 4:** Demographics of subjects with limb loss.

**Subj**.	**Age** **(yrs)**	**Sex**	**Time since** **amputation**	**Main prosthesis** **(Alt)**	**BP exper.** **level**	**MP exper.** **level**
LL1	37	F	37 years	Cosmetic	Low	High
LL2	23	F	22 years	None (MP)	None	Med
LL3	41	M	7 years	BP (MP)	Low	High

*MP, myoelectric prosthesis; BP, body-powered prosthesis*.

The set of assessments was built within the International Classification of Functioning, Disability and Health (ICF) framework. For each configuration, the experimental session consisted of brief training followed by functional testing and self-administered questionnaires on usability and pleasantness of the device tested. Configurations were presented in a pseudo-random order. In training, subjects partook in 30 min of grasping and manipulation practice, with objects of daily-living of different shapes, weights, and softness. To the authors' knowledge, standard tests of hand function that replicate work tasks and/or a work environment have not been developed. The performance evaluation was thus composed of the Assessment of Capacity for Myoelectric Control (ACMC) test and the Box and Blocks Test (BBT). For the ACMC, four tasks among six were chosen: setting the table, packing a suitcase, preparing a dessert, and organizing the mail. The order of the four tasks of the ACMC were also pseudo-randomized. The randomization of the ACMC tasks and prosthetic configurations was adjusted to ensure a balance between the order of configurations, order of tasks, and the number of times a particular configuration was matched with a particular task. Among the authors, we have four trained and certified raters, two of whom co-scored the test. The devices were also rated using the System Usability Scale (SUS). During all procedures, comments by subjects on tests and devices were gathered. Rest periods were included as needed both within and between sessions. Overall, the entire measurement session lasted about 2 h.

## 3. Results

### 3.1. Limb-Intact Subjects

Following training, in which subjects practised both control modes (voluntary-open and voluntary-close, VO and VC, respectively) and how to switch between them, subjects were free to choose with which control mode to start the ACMC test. Out of 20 trials (2 ACMC tests across 10 subjects) only in 1 instance, or 5%, did the subject opt to start in VO. They were allowed to switch as many times as desired during the test; the number of times subjects switched volitionally was not recorded. We requested that subjects announce when they accidentally switched between voluntary-open and voluntary-close control modes. The Hardy Configuration had a higher average accidental switch rate than the Compact Configuration (1.9 ± 2.1 and 0.8 ± 0.9 switches per ACMC test, respectively).

**Figure 6:**
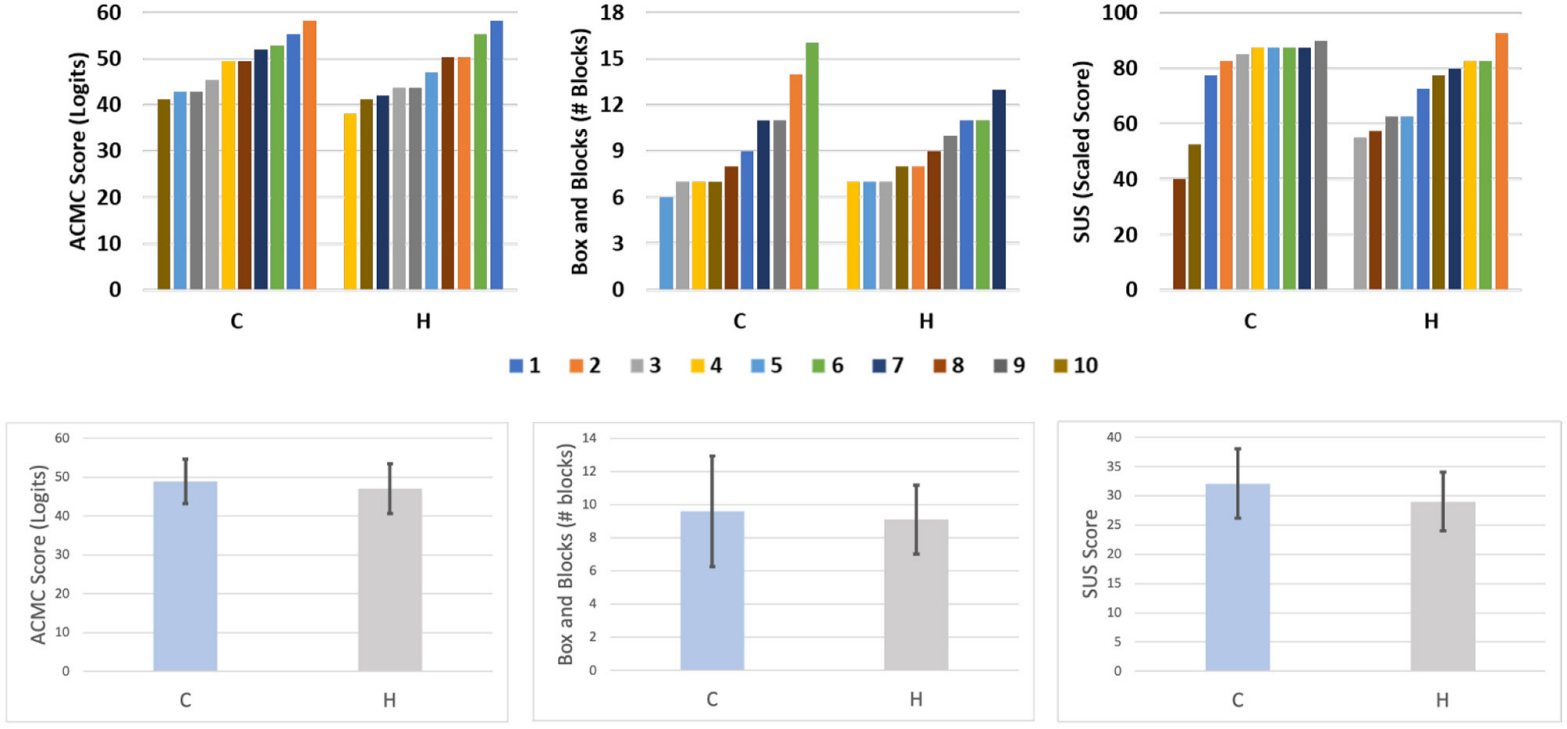
Results of limb-intact subjects on the ACMC, BBT, and SUS for each configuration presented individually, color-coded by subject, in the **top** row and in group form in the **bottom** row. Group results are presented as bar plot, the black bar denotes the standard deviation.

Plots showing the results of the three tests across limb-intact subjects are presented in Figure 6. Group results for these subjects are presented in Table 5. Immediately evident from both sets of charts is the extent to which the results of the two configurations overlap. The Compact Configuration, however, appears to perform slightly better on all three tests. The distribution of the results passes the Shapiro-Wilk Test for normality and thus means were compared using a student's *t*-test and no significant differences were found between Compact and Hardy configurations. Additionally, the difference between the SHPH-C and SHPH-H in the ACMC does not exceed the value of “minimum detectable change” of 2.5 logits.

**Table 5:** Limb-intact subject results.

	**Solution C**	**Solution H**
	**Mean ± standard deviation**	**Mean ± standard deviation**
ACMC	49 ± 5.73	47 ± 6.45
BBT	9.6 ± 3.34	9.1 ± 2.08
SUS	77.8 ± 6.89	72.5 ± 5.01

### 3.2. Subjects With Limb Loss

**Figure 7:**
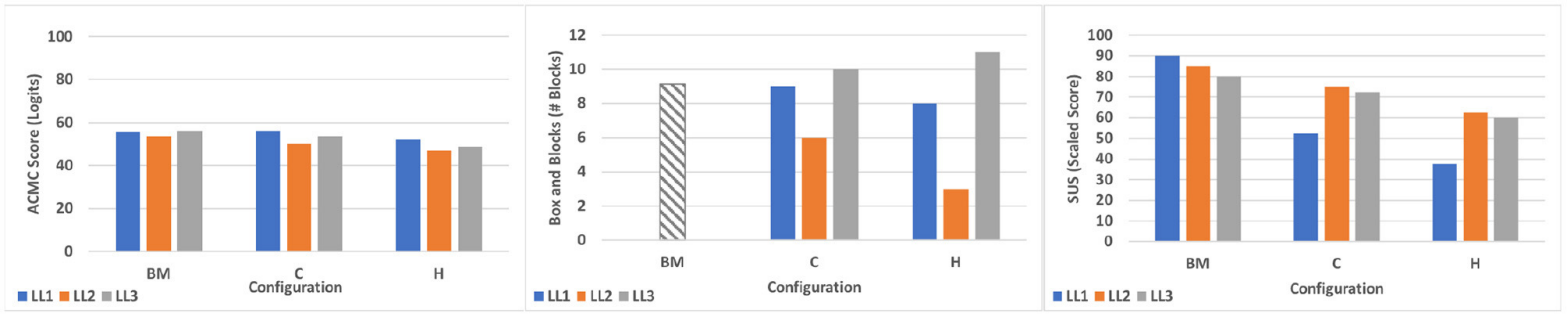
Results from subjects with limb loss testing the two hybrid configurations in comparison with the benchmark. Box and Blocks data of the benchmark are extracted from Godfrey et al. (2018).

**Figure 8:**
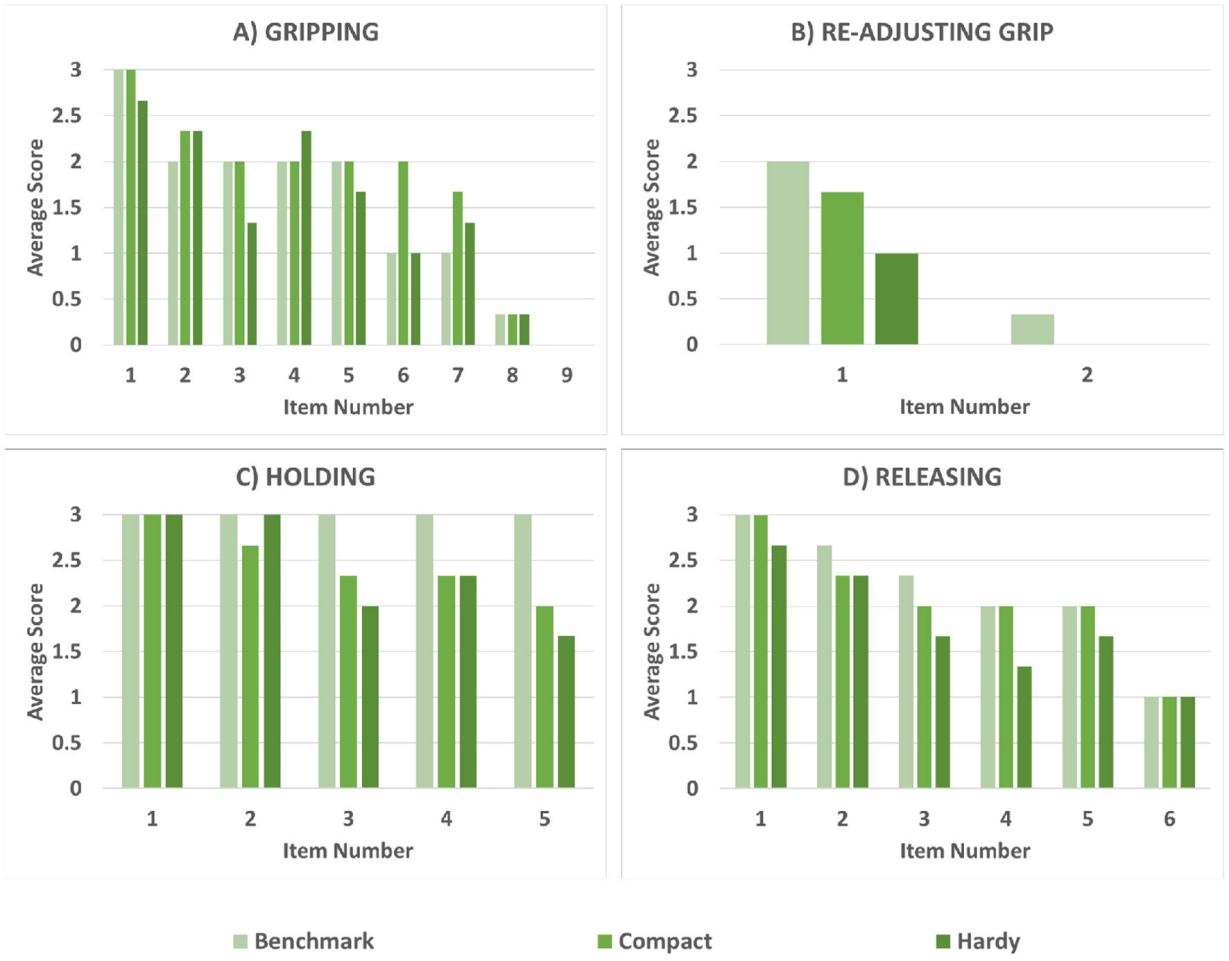
Average results of the three tested configurations for each ACMC item. The full list of items is reported in Table 8.

**Figure 9:**
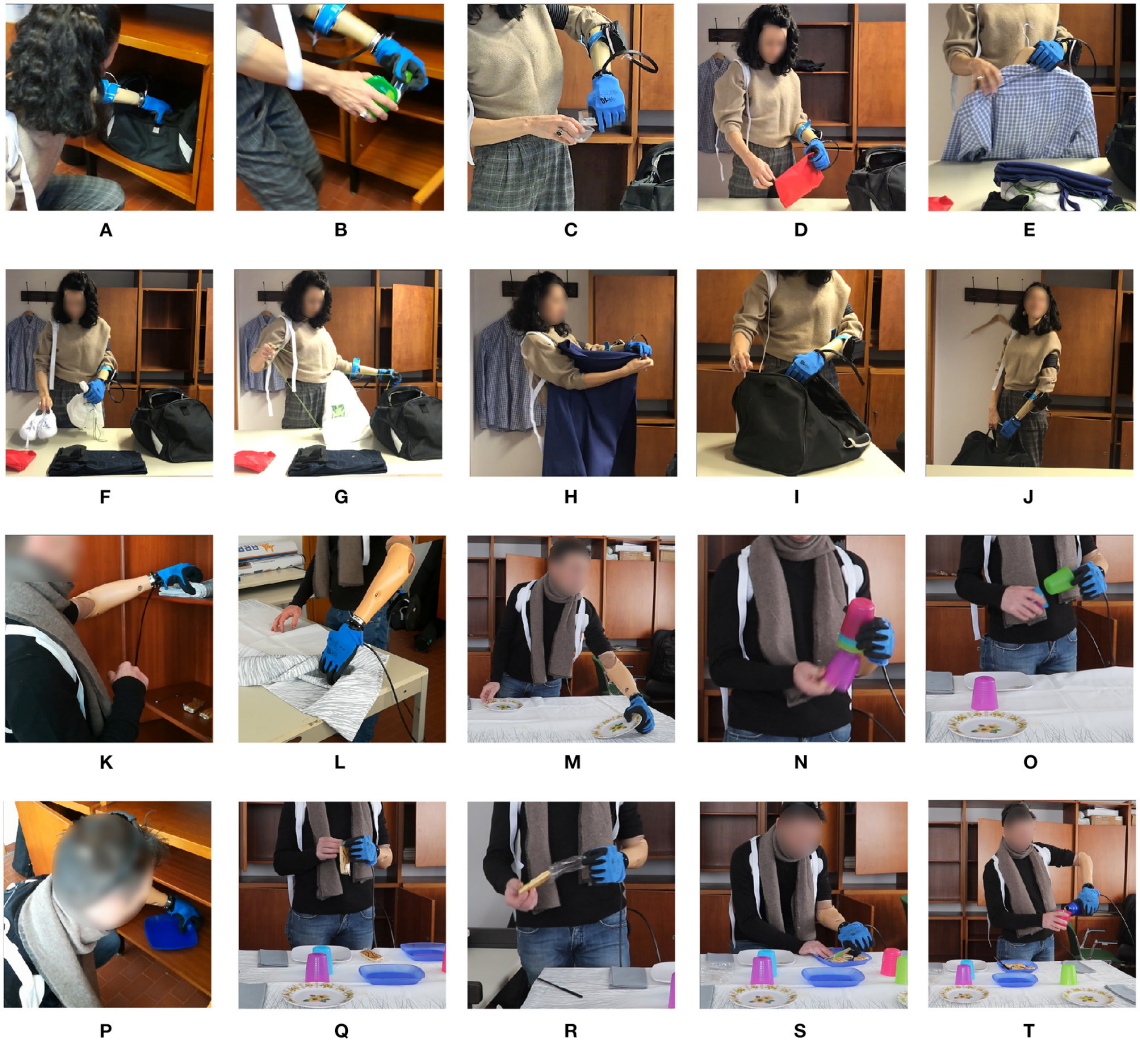
**(A–J)** are still images of the various activities performed in the ACMC “packing” task, while **(K–T)** are taken from the ACMC “setting the table” task. The subject is using the Compact Configuration in the packing task and the Hardy Configuration in the setting the table task.

Individual results from testing with subjects with limb loss can be found in Table 6 and Figure 7, while group results are presented in Table 7. As described in section 2, our primary objective outcome measure was the ACMC. All results fall into the same category of clinical interpretation and are classified as “Generally Capable,” which comprises scores between 46.7 and 57.1 logits. Furthermore, the minimum detectable change (MDC) for assessments performed by the same rater is 2.5 logits. In a pair-wise, within-subject comparison of the scores, most fall outside of this range, except for LL1 SHPH-C and SHP. Between subjects, pair-wise comparisons are less than the MDC for the SHP (LL1 and LL3, and LL1 and LL2) and the SHPH-H (LL2 and LL3). Taking this into consideration, subjects LL2 and LL3 exhibited the most control capacity with the SHP followed by the SHPH-C and finally the SHPH-H. LL1 performed equivalently with the SHPH-C and the SHP and less well with the SHPH-H. Figure 8 shows the breakdown of the scores of the ACMC for the three subjects with limb loss. Please note: the test is not designed to be evaluated based on individual items; however, these are presented to provide a more complete picture of the comparison between control modes. The items in each category increase in difficulty (along the x-axis); this increase is reflected in the overall decrease in scores along this axis. Across the 66 items (22 items per test and three subjects), SHPH-H control capacity was rated lower than that with the SHPH-C 32% of the time, equal 59% of the time, and superior 9% of the time. Capacity of control of the SHPH-C was more similar to that of the SHP, with control rated inferior, equal, and superior 20, 70, and 10% of the time, respectively. Images of two subjects with limb loss performing various activities from the ACMC tasks with each SHPH Configuration can be found in Figure 9.

**Table 6:** Limb loss subject results.

	**LL1**			**LL2**			**LL3**		
	**BM**	**C**	**H**	**BM**	**C**	**H**	**BM**	**C**	**H**
ACMC	55.4	56.3	51.9	53.7	50.3	47	56.3	53.7	48.7
BBT	-	9	8	-	6	3	-	10	11
SUS	90	52.5	37.5	85	75	62.5	80	72.5	60

*The results of the three subjects with limb loss are presented as raw data*.

**Table 7:** Limb loss subjects average results.

	**Median BM**	**Median C**	**Median H**	**Mean BM**	**Mean C**	**Mean H**
ACMC	55.4	53.7	48.7	55.1	53.4	49.2
BBT	11◇	9	8	9.6◇	8.3	7.3
SUS	85	72.5	60	85	66.6	53.3

◇ *Data extracted from Godfrey et al. (2018)*.

**Table 8:** Description of the 22 items evaluated by ACMC.

A	GRIPPING
1	With Support
2	Power Grip Without Support
3	Precision Grip Without Support
4	Appropriate Force
5	In Different Positions
6	Timing
7	Coordinating Both Hands
8	Without Visual Feedback
9	Appropriate Force Without Visual Feedback
B	RE-ADJUSTING GRIP
1	Repetitive Grip and Release
2	Repetitive Grip and Release Without Visual Feedback
C	HOLDING
1	With Support
2	Without Support
3	In Motion
4	Without Visual Feedback
5	In Motion, Without Visual Feedback
D	RELEASING
1	With Support
2	Without Support
3	In Different Positions
4	Timing
5	Coordinating Both Hands
6	Without Visual Feedback

The SUS was used as a primary subjective outcome measure, while the BBT was a secondary outcome measure in evaluating the SHPH configurations and SHP. On the SUS, the benchmark achieved the highest scores for all three subjects, followed by the SHPH-C and then the SHPH-H. Results were more mixed for the BBT. Comparing the two SHPH configurations: LL1 and LL3 moved nearly the same number of blocks across configurations (9 and 8 blocks for LL1 and 10 and 11 blocks for LL2 for SHPH-C and SHPH-H, respectively). Both of these subjects were near the mean for the SHP (9.6) found in a previous study with 9 subjects with limb loss (Godfrey et al., 2018). Subject LL2 was markedly below this value, performing better with the SHPH-C than the SHPH-H (6 and 3 blocks, respectively).

Anecdotally, we asked subjects to report which configuration among the two SHPH they preferred overall and additionally asked which configuration's control they preferred. Note: the Compact Configuration used the figure-of-nine harness (single shoulder), while the Hardy Configuration used the figure-of-eight harness (double shoulder). Two subjects (1 and 3) preferred the Hardy Configuration in terms of control and two subjects (1 and 2) preferred the Compact Configuration overall. All subjects chose to start in VO mode for both configurations. Finally, subjects had 1, 4, and 2 involuntary switches with the Compact Configuration compared with 1, 2, and 0 with the Hardy Configuration.

## 4. Discussion

The SHPH-C and SHPH-H were preliminarily validated with intact-limb subjects before testing with subjects with limb loss. Among these subjects, the Compact Configuration slightly outperformed the Hardy Configuration on all three outcome measures (ACMC, BBT, SUS), but not significantly so. Additionally, it is worth noting the clustering of SUS scores, in particular for the Compact Configuration. These scores approach the upper limit of the SUS, and the clustering could suggest either a ceiling effect of the measure or simply agreement among those who rated the configurations more positively. The Compact configuration has more weight distal to the torso but is less cumbersome in terms of donning and wearing (using a figure-of-nine harness and socket, with nothing else worn on the body). One of the limitations of the study was that the Hardy Configuration was noticeably slower than the other configurations due to friction in the Bowden cable limiting transmission of motion from the motor to the hand. It is difficult to infer the impact of this issue: one might expect a significantly slower hand to show drastically decreased performance in the BBT, while this was not the case. Slower movement, especially among subjects with limited training could have resulted in more precise movements as they allowed the subject more time to adjust the grasp and hand position during closure. This phenomenon could be reflected in the BBT results for the Hardy Configuration compared to the Compact: the slower hand could mask some of the variability seen between subjects with the Compact Configuration. This decreased speed could nevertheless have been a contributing factor in the less-favorable subjective ratings.

**Figure 10:**
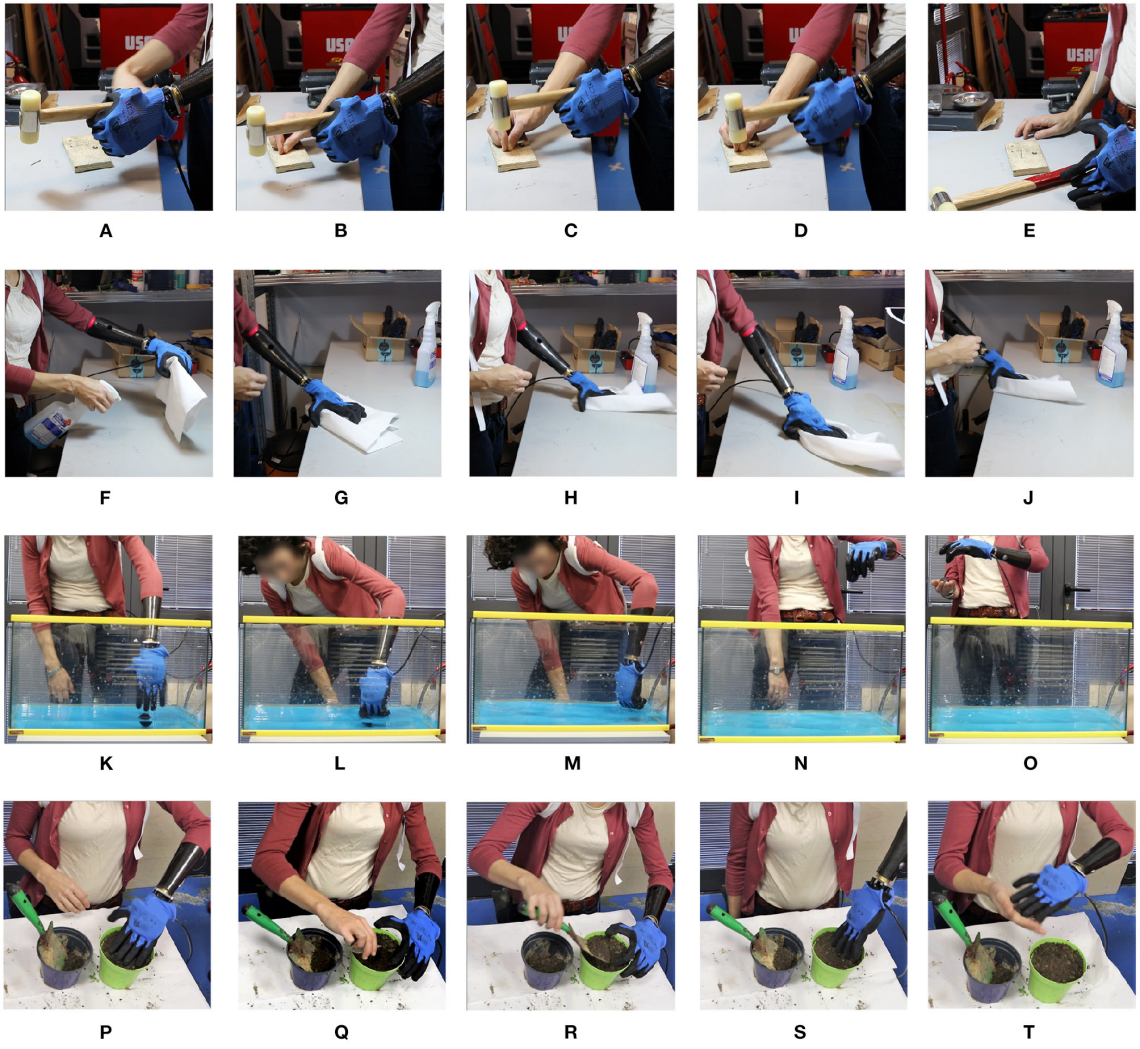
Still images from various work tasks performed by a subject with limb-loss using the Hardy Configuration. From top to bottom the tasks are: hammering a nail **(A–E)**, wiping down a work surface **(F–J)**, retrieving an item in water **(K–O)**, and planting seeds **(P–T)**.

In the comparison between myoelectrical- and body-controlled electric devices, the main objective outcome measure of this study, the ACMC, showed subjects with limb loss were able to perform at the “Generally Capable” level with all three devices tested. These subjects had more experience with myoelectric control compared to body-powered and all had some exposure to the SHP in the past. Despite this advantage, they were able to reach the same clinical level with the SHPH after limited training, and in one instance (LL1, SHPH-C) reach a score equivalent to that with the SHP. The limb-intact group shows a much wider range of results on the ACMC, with control of the SHPH-C being rated “Somewhat Capable” 4 times (5 times for the SHPH-H), 5 times “Generally Capable” (4 times for the SHPH-H), and 1 time “Extremely Capable” for each configuration. The notable difference in the range of scores between the limb-intact and limb-loss groups is reasonable given the different levels of exposure to prosthetic technology. It is to be expected that longer-term users could leverage their experience, both with other prostheses and control methods as well as with the SHP, to perform reasonably well on the ACMC while the limb-intact group would acquire mastery of the prosthesis and its control at different rates. As mentioned earlier, the ACMC individual item scores should be examined with care as the test is designed to be taken as a whole. These data can still provide interesting insight: for example, it is reasonable to infer that subjects' performance, in particular in challenging tasks such as grasping or holding without visual feedback and/or in motion, relates not only to the subject's capacity in performing a particular task but also to their confidence in that capacity. It is worth noting that all three subjects reached the maximum across all “Holding” items with the SHP but only one subject did with the SHPH-H and none with the SHPH-C. This could be due to the limited exposure to body-powered prosthesis control and a resultant lack of confidence in using it. Furthermore, the individual items show that the slightly lower performance with the SHPH-H in comparison with the SHPH-C is not due to a major decrease in performance in any one specific category but rather to a slight underperformance across the range of items. Additionally, to our knowledge, there are no standardized tests that replicate a work environment. As the SHPH class of devices was designed in large part for work environments and manual tasks, one of the subjects with limb-loss performed several relevant tasks following study completion. Still images from these tasks are presented in Figure 10 and consist of hammering a nail, submerging the hand in water to retrieve an item, wiping down a work surface, and planting seeds. These tasks were chosen because they require high forces and/or recreate environments and tasks that are typically challenging for prostheses, including exposure to dirt and immersion in water. Videos of the subject completing the various tasks are available as a multimedia attachment to the present work. While not the result of a standard test, these successful attempts support the idea that the SHPH could be a high-functioning prosthetic device in these environments. In the future, this aspect will be explored more in-depth.

In future studies, one could study the trade-off in comfort between distal weight, that over the long-term would likely induce fatigue, and simplicity of donning and wearing by providing subjects with limb loss longer exposure to the Compact and Hardy Configurations. To minimize the likelihood of a training effect, beyond randomizing the order, similar comparisons in the future could lengthen the training session prior to testing the first configuration or use a proxy to increase training time. For example, future SHPH tests could include an initial training session with the myoelectrical controlled SHP to familiarize the subject with the terminal device and/or a standard body-powered prosthesis to better internalize the control scheme. Since both configurations performed similarly well on the objective outcome measures, future work will also focus on improving the mechanical implementation of the Hardy Configuration. In particular, as mentioned above, optimizing the speed of the terminal device, streamlining donning, and adjusting the configuration to be less bulky would likely improve device performance and acceptance.

## 5. Conclusions

In this work, we presented and assessed two different prototypes of the SoftHand Pro-Hybrid that integrate the use of soft robotic technologies with non-EMG based controls. In a preliminary validation with subjects without limb loss, both Configurations performed well, with the Compact Configuration slightly outperforming the Hardy in both objective and subjective tests. We then tested both of these Configurations with three subjects with limb loss and compared these results to the myoelectrically controlled SHP, used as a benchmark. This group had similar results with the Compact slightly outperforming the Hardy configuration on the ACMC and the benchmark outperforming both in two out of three subjects. Across configurations, however, all three subjects were rated as “Generally Capable” on the ACMC with all three configurations. Despite the limited number of subjects, this pilot study suggests the reliability of the SHPH configurations and the possibility to use hybrid solutions as a valid alternative to myoelectric control, especially in challenging environments. Improvements in the speed of the Hardy Configuration may improve both subjective and objective evaluations. Additionally, it is possible that further exposure to and more intensive training with the SHPH configurations could help improve performance. Encouraging results also open new avenues for the design of a different class of prosthetic device (i.e., partial hands) based on a similar hybrid method.

## Data Availability Statement

The original contributions presented in the study are included in the article/Supplementary Material, further inquiries can be directed to the corresponding author/s.

## Ethics Statement

The studies involving human participants were reviewed and approved by Comitato Etico di Area Vasta Nord Ovest, CEAVNO. The patients/participants provided their written informed consent to participate in this study.

## Author Contributions

SBG and FF performed the experiments. SBG, CP, GG, and MC performed the data analysis and designed the experimental setup. All authors contributed to writing the manuscript and designed the study.

## Funding

This project has received funding from the European Union's Horizon 2020 ERC programme under the Grant Agreement No. 810346 (Natural Bionics) and by the European Research Council under the Proof of Concept Grant SoftHand Pro-H (No. 727536). The content of this publication is the sole responsibility of the authors. The European Commission or its services cannot be held responsible for any use that may be made of the information it contains.

## Conflict of Interest

AB, MC, and GG are cofounders and shareholders of qbrobotics s.r.l., a company producing robotic hands and components of the SoftHand Pro used in the experiments reported in this paper. The remaining authors declare that the research was conducted in the absence of any commercial or financial relationships that could be construed as a potential conflict of interest.

## Publisher's Note

All claims expressed in this article are solely those of the authors and do not necessarily represent those of their affiliated organizations, or those of the publisher, the editors and the reviewers. Any product that may be evaluated in this article, or claim that may be made by its manufacturer, is not guaranteed or endorsed by the publisher.
